# KCNN2 Mutation in Pediatric Tremor Myoclonus Dystonia Syndrome with Electrophysiological Evaluation

**DOI:** 10.5334/tohm.668

**Published:** 2022-01-24

**Authors:** Bennett Lavenstein, Patrick McGurrin, Sanaz Attaripour, Felipe Vial, Mark Hallett

**Affiliations:** 1 Children’s National Health System, Washington DC, USA; 2 Human Motor Control Section, National Institute of Neurological Disorders and Stroke, National Institutes of Health, Bethesda, MD, USA; 3 Department of Neurology, University of California, Irvine. 200 S. Manchester Ave., Ste 206, Orange, CA, 92868, USA; 4 Facultad de Medicina Clínica Alemana Universidad del Desarrollo, Santiago, Chile

**Keywords:** Myoclonus, Genetics, Physiology

## Abstract

**Background:**

Here we combine clinical, electrophysiological, and genetic findings to phenotype an unusual childhood movement disorder in a patient with a rare form of KCNN2 mutation.

**Case Report:**

A 10-year-old male presented with a clinical syndrome of tremor and myoclonus. Electrophysiology demonstrated muscle activity indicative of myoclonus dystonia, an observation that was not appreciated clinically. Genetic testing revealed an abnormality in the KCNN 2 gene, not present in the parents, known to cause dystonia, as the etiology.

**Discussion:**

The value of utilizing noninvasive, electrophysiological recording in pediatric movement disorders expands the precision of diagnosis, potentially informing treatment when correlated with clinical and genetic findings.

## Introduction

The evaluation of patients with involuntary jerky movements is always a challenge. A correct characterization of the phenomenology requires differentiating the pattern and duration of muscle contractions in a millisecond range. This first step is crucial to narrow down the myriad of possible diagnosis.

We are presenting here a case in which we use electrophysiology to make a syndromic diagnosis and then whole exome sequencing to make an etiological diagnosis.

## Case Report

A 10-year-old male presented to the Human Motor Control Section at the National Institutes of Health with a complaint of “shaking of his hands,” exaggerated startle, low muscle tone, and lack of coordination. By observation, he was noted to have abnormal hand movements early in his life. These were described as “jerky” movements, said by his parents to be present when handling small toys and other actions around age 3. With more motoric activities these movements became more bothersome, and at the time of presentation in middle childhood, writing was particularly difficult. He noted some truncal movements that both he and his parents described as “jerky.” Movements were worse with stress or when he was cold. Parents reported that he was startled easily and extensively. He was previously noted to have weak core muscles by a physical therapist, as well as intermittent toe walking that was more prevalent at a younger age. He was startled by bodily touch, loud sounds, and minor stimuli. Parents reported difficulty standing on one foot, inability to hop, to do jumping jacks easily, or walk down a flight of stairs with alternate feet.

The patient was the product of a full-term and uncomplicated pregnancy, normal labor and delivery (normal Apgar scores), and was discharged home without complications. He had a history of delay in developmental milestones, walking at age 18 months, and speech starting at 3 years of age. Fine motor and social milestones were described as normal.

Prior history also revealed middle ear infections, placement of ET tubes, history of pneumonia × 2, and a history of childhood cyclical vomiting. These conditions subsequently resolved. He has 3 siblings who are in good health, as are his parents. The family has a history of migraine (mother), cystic fibrosis carrier (mother), and Parkinson’s Disease (great grandfather).

A general review of systems was unremarkable. Neurologic examination disclosed an awake, oriented, and appropriate appearing 10-year-old, well-nourished, slightly asthenic male with slight head flexed posture. Cranial nerve evaluation was unremarkable with normal ocular motility and no nystagmus. Full range of head and neck motions was present. Motor examination revealed diminished muscle bulk throughout but without weakness. Muscle tone was normal. He was on no medications at the time of evaluation. Previous diagnostic studies revealed normal brain MRI and normal EEG (awake). Laboratory studies were unremarkable.

Clinically, both postural and kinetic tremors were present. Myoclonic jerks in the eyelids, trunk, and bilateral upper extremities were present at rest and with posture (see ***Video 1***). Movements were also present in response to any stimulus, including touching the patient, moderately loud sounds, and eliciting deep tendon reflexes. Finger tapping and handwriting were interrupted by myoclonus. Tandem gait revealed impairment within the first few steps, which did not subsequently correct or improve (see ***Video 2***). Abnormal postures indicative of dystonia were not present. Sensory examination was normal to all modalities. No pathologic reflexes were elicited. This phenomenology was consistent with tremor and myoclonus, thought possibly to be cortical myoclonus and cortical tremor.

**Video 1:** **Clinical Assessment.** Video depicting the bilateral upper extremities during wingbeat, as well as during a finger tapping task.

**Video 2:** **Tandem Gait.** Patient performing a tandem gait task.

## Electrophysiology

To characterize the movements, we performed an electrophysiological study. Sticky surface electromyographic (EMG) electrodes were attached to bilateral extensor carpi radialis (ECR), flexor carpi radialis (FCR), biceps, and triceps. Electroencephalographic (EEG) was recorded from the bilateral sensory-motor cortex.

**Figure 1:**
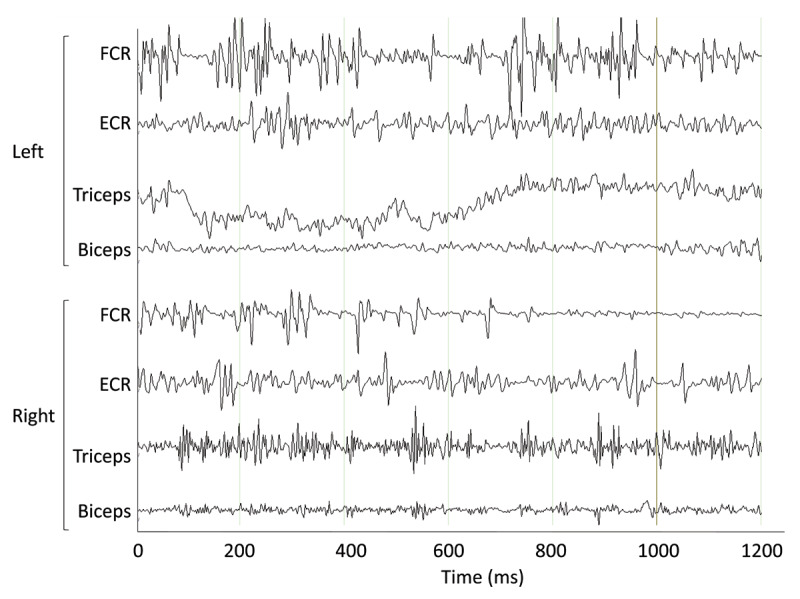
Electromyography recorded from bilateral upper extremities during posture with arms supported.

EMG revealed irregular bursting activity, with both short and long bursts. Short duration (15–20 ms) biphasic potentials with consistent shape were present during the recordings, without clear agonist-antagonist co-activation. Longer bursts (50–200 ms) were also present and appeared responsible for the involuntary movements. There was no observed correlation between EEG and EMG (***Figure 1***). The tremor seen clinically was not observed on the EMG to have a consistent burst pattern or frequency. Performing a fast fourier transform to convert the time series into the frequency domain did not reveal any clear frequency peaks. Additionally, weight loading with two pounds did not reveal any change in the characteristics of the EMG activity.

To assess cortical hyperexcitability, we recorded somatosensory evoked potentials (SSEPs) using median nerve stimulation with an intensity set to 3 times the sensory threshold, pulse width 0.5 ms, frequency 3 Hz. SSEP and C reflex testing revealed an SSEP with normal latency and amplitude. No signs of cortical hyperexcitability were noted. In addition, we performed a back-averaging analysis. We used left and right biceps to perform this analysis, as we felt that the other muscles recorded did not provide a sufficient number of movements for calculation. Back-averaging to both left and right biceps did not reveal a preceding cortical component.

**Figure 2:**
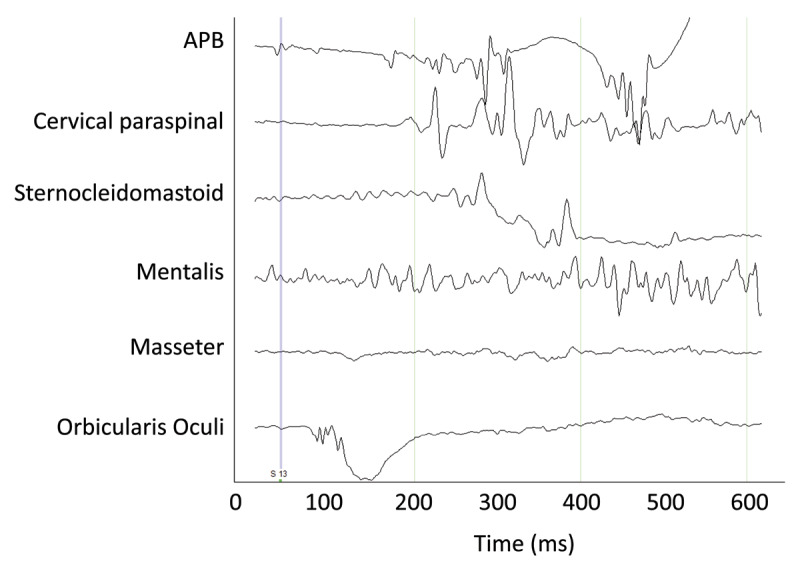
Electromyography during the auditory startle testing. The vertical line indicates the timing of the auditory stimuli for a single trial.

To assess what we thought clinically to be a startle response, a 1500 Hz, 120 dB tone was delivered with headphones. EMG was recorded from left orbicularis oculi (OO), masseter, mentalis, sternocleidomastoid, cervical paraspinal, and abductor pollicis brevis (APB). A total of 14 stimuli were presented, with an interstimulus interval of ~1 minute. There was a large reaction after the first stimulus. The OO response showed an auditory blink reflex at a normal latency of 30 ms. Subsequent EMG responses due to this first stimulus were disorganized and occurred more than 140 ms after the stimulus (***Figure 2***), and thus were considered to be an exaggerated response, but not a physiological startle response as it did not match the expected pattern of EMG activation. Following the first stimulus, there was only contraction of the OO with a normal 30 ms latency. From this information we concluded that no exaggerated startle reflex was present.

Considering the lack of evidence for cortical excitability, together with the presence of long EMG bursts, the electrophysiology diagnosis was made to be dystonic myoclonus. The shorter duration potentials were carefully reviewed and given their consistent shape and duration were considered to be motor units.

## Genetic study

Whole exome sequencing identified a pathogenic heterozygous variant considered disease-causing in the KCNN2 {c.1084 G>A) gene. Whole exome sequencing of the parents did not disclose this KCNN2 variant. Whole genome testing was not performed.

## Discussion

We performed an electrophysiological study to evaluate a 10-year-old boy who presented with concerns for tremor, jerky movements, and impaired ability to carry out motor tasks, but no obvious abnormal posturing. Genetic testing with whole exome sequencing revealed an abnormality in the KCNN2 gene not noted to be present in his parents, supporting evidence for a syndrome of myoclonus dystonia presenting in a sporadic form.

While the clinical findings of myoclonus were readily apparent, electrophysiology was done to further refine the phenomenology. The burst pattern and duration of the EMG was indicative of myoclonus dystonia, an observation which was not suspected on clinical examination. In addition, the tremor seen clinically was revealed to be intermittent and without a clear, consistent burst pattern, and was likely driven by these longer bursts seen on the EMG.

Advances in sequencing have improved the evaluation of genetic movement disorders. With rare and atypical presentations of neurologic conditions, genetic testing yields a diagnosis in about 40% of cases and in some instances up to 90%.

The Potassium intermediate/small conductance calcium-activated channel, subfamily N, member 2 (KCNN2) is a protein encoded by the KCNN2 gene and thought to regulate neuronal excitability by moderating small conductance calcium-activated channels (SK2) [1,2,3]. KCNN2 is highly expressed in the cerebellum and there is overlap with gene co-regulation analysis revealing similarity between KCNN2 and other genes implicated in myoclonus.

KCNN2 mutations may produce multiple phenotypical features similar to those of other mutations that are deleterious. In the more common dominant form, the clinical features may include myoclonic jerks, dystonia of the neck, trunk, upper limbs, psychiatric symptoms, and alcohol responsiveness. Balint et al. have recently reported a family pedigree of eight persons with a phenotype resembling myoclonus dystonia over three generations with a novel missense variant in the KCNN2 gene [4]. They stressed the autosomal dominant nature of this disorder and its overlap with the well-described myoclonus dystonia syndrome related to the epsilon-sarcoglycan gene mutation. By comparison, in the patient we report here tremor without abnormal postures was the predominant finding and myoclonus was associated and observed phenotypically. This distinguishes this phenotypical presentation from the previously reported series where abnormal posturing was the cardinal feature.

Variants of KCNN2 mutations are rare, as is the recessive form, that may occur in 1% [5]. The rodent model of this variant is associated with stiff gait, hunched posture, and tremors starting from 3 weeks of age [6]. To our knowledge this phenotype has not been well described in humans.

Here we present clinical and genetic findings in a patient with a rare sporadic form of KCNN2 who presented with tremor and myoclonus in childhood. The electrophysiology makes clear the clinical-genetic correlation which allows for a diagnosis that is defined electrophysiologically distinctly different than the well-described phenotypical myoclonus dystonia syndrome due to mutation in the epsilon sarcooglycan gene. Clinical neurophysiology is valuable in extending the phenotypical description of involuntary movements making more exact diagnoses possible.
